# Multi-driver and multi-scale assessment of vine community structure and composition across a complex tropical environmental matrix

**DOI:** 10.1371/journal.pone.0215274

**Published:** 2019-05-10

**Authors:** Diana L. Delgado, Carla Restrepo

**Affiliations:** 1 Department of Biology, University of Puerto Rico-Bayamón, Bayamón, Puerto Rico; 2 Department of Biology, University of Puerto Rico-Rio Piedras, San Juan, Puerto Rico; Aarhus University, DENMARK

## Abstract

Ecological communities are structured by multiple processes operating at multiple scales yet understanding the scale-dependency of these processes remains an open challenge. This might be particularly true for parasites, for which biotic rather than abiotic processes may play a primary role in structuring communities. Focusing on vines, a group of structural parasites that gain access to the canopy using different climbing mechanisms, we examined the influence of abiotic factors in tandem with *host-parasite* and *parasite-parasite* interactions in the assembly of tropical vine communities. Two synthetic variables, namely *Climate1* and landscape *Variety*, were consistently important in explaining variation in species richness and diversity, as well as species composition, but their importance varied with scale. Whereas *Climate1* summarizes the largest variability among climatic variables, landscape *Variety* expresses landscape heterogeneity within a neighborhood. Significant patterns of species co-occurrences suggest that *vine-vine* interactions also contribute to vine community assembly. Our results may be critical to understand vine proliferation and help design management strategies for their control.

## Introduction

Ecological communities are structured by abiotic and biotic processes operating at multiple scales [1], yet understanding their scale-dependency remains an open challenge [2, 3]. It is generally acknowledged that these processes operate in a hierarchical fashion to determine species’ distributions and ultimately, the structure of ecological communities (e.g., [4, 5], but see [6]). One group of species for which this hierarchy of processes may not apply includes a broad spectrum of symbionts—ranging from mutualistic to parasitic species [7, 8]. Among parasites, biotic processes such as *host-parasite* interactions may be more important than abiotic processes in determining the structure of parasite communities [7–9]. Albeit less studied, *parasite-parasite* interactions may play a similar role [7, 10]. To date, however, few studies have examined the role of abiotic factors in tandem with *host-parasite* and *parasite-parasite* interactions in community assembly [11, 12].

Host-parasite interactions mediate the structure of parasite communities through multiple mechanisms. Hosts may influence colonization [13] and transmission [14] of parasites, deploy defenses to limit the damage by parasites [15], and control the energy and nutrient content of tissues that parasites use to reproduce [16]. On the other hand, parasite-parasite interactions can influence the structure of parasite communities through mechanisms involving competition and facilitation [17]. Co-occurring parasites may engage in competitive or facilitative interactions that may have different fitness outcomes for the interacting species [18, 19]. Similarly, hosts’ immune-mediated responses towards one parasite may increase susceptibility to infection by other parasites [20].

A unique host-parasite system that may help understand the role of abiotic and biotic processes in structuring communities of parasites includes climbing plants and their hosts. Climbing plants are commonly classified as structural parasites due to their dependence on other plants for support which often results in negative impacts on the latter [21]. Climbing plants exhibit a variety of climbing mechanisms and growth strategies that allow them to gain access to areas with abundant light. At the scale of individual hosts, the presence of climbing plants can result in the overshadowing of plant canopies or an increase in below-ground competition that results in reduced growth and host death [22, 23]. At the scale of forest stands, climbing plants can reduce biomass and change the soil biogeochemistry [24] with implications for global carbon budgets [25, 26]. On the practical side, understanding the role of abiotic and biotic processes in structuring communities of climbing plants may help us gain a better understanding of the well-documented increase in climbing plant abundance in different regions around the world [27–29].

Climbing plants form communities with diffuse or discrete boundaries [30] that vary in taxonomic and functional diversity [31, 32]. Woody climbing plants or lianas often form diffuse communities [30] in which liana diversity increases with annual precipitation or precipitation seasonality [33, 34], and soil fertility [35, 36]. However, it has also been shown that liana diversity increases with host abundance and diversity [37, 38]. The latter observation suggests that *host-climber* interactions can influence the composition and structure of climbing plant communities. In particular, host structural characteristics and parasite climbing mechanism may play a critical role in community assembly [32, 39, 40]. Additionally, young lianas can grow over old and large lianas [41], suggesting that *climber-climber* interactions may also play a role in the assembly of liana communities.

Here we develop a novel multi-driver and multi-scale approach that focuses on herbaceous climbing plants or vines to investigate the relative importance of abiotic and biotic processes in community assembly. Our approach takes advantage of the increasing presence of invasive native and alien species in agricultural and post-agricultural landscapes [42, 43] and the compact organization of these communities that allows the precise mapping of vine patches from remotely sensed data [44]. We combine field sampling of vine patches across a complex environmental gradient with spatially explicit abiotic and biotic variables describing climatic, edaphic and topographic conditions, as well as host characteristics at three spatial scales. We hypothesized that due to the parasitic nature of vines, biotic factors had a stronger influence on the diversity and composition of vine communities than abiotic factors. We further hypothesized that the importance of the abiotic and biotic variables influencing vine diversity and composition varied with scale. We asked three specific questions. First, to what extent does a small group of abiotic and biotic variables explain regional patterns of alpha and beta diversity among structural parasites? Second, does a variation in spatial scale provide insights into the factors driving variation in parasite diversity and composition at regional scales? Lastly, what is the importance of species co-occurrences in structuring vine communities?

## Materials and methods

### Study area

**Fig 1:**
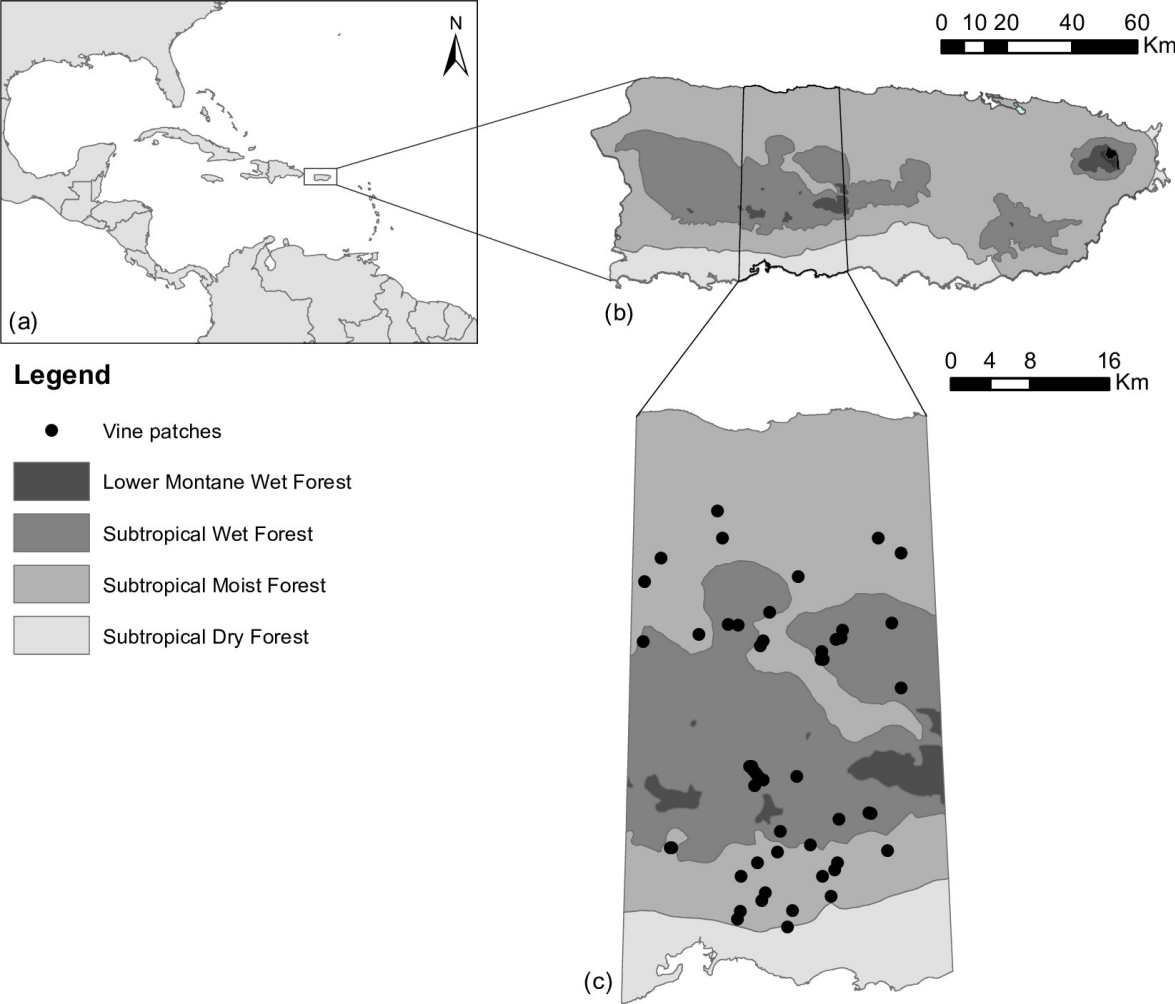
Map of the study area. Location of (a) the Island of Puerto Rico (Latitude 18.2208, Longitude -66.5901), (b) study region within Puerto Rico, and (c) vine patches sampled in the study region.

Our study took place in a region extending from the northern to the southern coast of central Puerto Rico (total area 1,763 km^2^ or equivalently 20% of the island; Fig 1). This region encompasses a diverse array of bioclimatic, geologic, and edaphic conditions as well as land uses (Table A in S1 File). The latter includes small patches of primary forest, extensive areas of secondary forest of various ages, active coffee plantations, and pastures [45]. In Puerto Rico vines form compact communities—hereafter vine patches–that cover ~3% (49.5 km^2^) of our study area [44]. The increasing proliferation of vines most likely is the result of the decline of an otherwise thriving agricultural economy [46] and the repeated introduction of numerous alien vine species with agricultural value [47].

### Sampling design

Between July 2012 –April 2013, we sampled 51 vine patches to determine vine abundance and composition based on a variant of the point-intercept method ([48]; Fig 1). The patches were randomly selected from a map depicting all vine patches in our study area (area ≥ 75 m2; [44]). The vine patches map was based on the analyses of high-resolution aerial photographs (taken by 3001 Inc. between November 2006—March 2007) with IMAGINE Objective (Hexagon Geospatial). The IMAGINE Objective software mimics the visual processing capability of the human eye in order to extract features from images based on spectral data, size, shape, texture. and shadows. We trained the software by providing at least 75 samples of vine cover areas per photograph. We also provided the same number of samples of other types of vegetation cover to help the software differentiate between the different types of vegetation and provided a more accurate classification. The resulting vectorial map of vine patches was revised and manually edited to generate a final map of vine patches [44]. The centroid coordinates of the selected vine patches were uploaded into a hand-held GPS (Trimble Geo XM 2005 series) to locate the patches in the field. Observed discrepancies between the mapped and current vine patch locations led us to collect new coordinates for the nearest vine patch. In the field, most of our patches occurred in public areas where permission for access and collection was not needed, but in the instances that the vine patches occurred in private property, we obtained verbal permission from the owners of the properties.

A preliminary sampling of ten patches allowed us to assess the effectiveness of the method to quantify vine abundance and to determine the sampling effort for patches of varying size. At each patch, we randomly selected points ≥1 m apart and introduced a sampling pole (91.4 cm long and 2.5 cm in diameter) that ran perpendicularly through the top layer of the patch. All live (green) vine stems and leaves that touched the terminal 10 cm of the sampling pole were identified to the species level in order to sample vines species growing at different levels in the vine patch. Based on our preliminary sampling, we determined that 12 (small patches; 5–10 m radius) and 20 (large parches; >10 m radius) sampling points were sufficient to capture most, if not all the vine species present within a vine patch. Our sampling strategy allowed us to account for the irregular shapes and the 3-D structure of vine patches resulting from vines growing on top of each other and existing structures of different heights and forms. All the vine species present in a patch were collected and identified to the species level with plant nomenclature following Axelrod [49]. In addition to taxonomic affiliation, vine species were classified into different functional groups based on their geographic origin (i.e., native or alien) and climbing mechanism (i.e., twining, tendrils, aerial roots, scandent/sarmentous; [50]).

### Vine relative abundance and density

We used the aforementioned data to calculate the abundance (*A*_*s*_) and density (*D*_*s*_) of the *s*^th^ vine species present in a patch according to Eq 1 and Eq 2.

$$A_{s}=\frac{p_{s}}{n}$$ (Eq 1)

$$D_{s}=\sum_{i=1}^{n}\frac{q_{s,i}}{m}$$ (Eq 2)

In Eq 1, *p*_*s*_, indicates the number of sampling points in which species’ *s* green stems or leaves touched the sampling pole and *n* is the total number of sampling points within a vine patch. In Eq 2, *q*_*s*,*i*_ the number of green stems or leaves of species’ *s* that touched the pole *i*, and *m* is the total number of green stems or leaves of all vine species that touched all poles within a vine patch. We used a similar approach to calculate the abundance of the *f*^th^ vine functional group whether defined by the climbing mechanism (*A*_*fc*_ and *D*_*fc*_) or geographic origin (*A*_*fg*_ and *D*_*fg*_) of vine species.

### Multi-driver and multi-scale assessment of vine diversity

We used spatially-explicit topographic, climatic, edaphic, and land cover data to investigate the role of abiotic and biotic variables in structuring vine communities at multiple scales. The data had different origins, was of different types, and had different resolutions (Table 1). Thus, our first task was to re-scale an initial set of four variables to match a 90-m resolution Digital Elevation Model (DEM). Our second task was to derive new variables that could describe the abiotic and biotic conditions of our study area (Tables A and B in S2 File).

**Table 1:** Biophysical variables used to characterize the vine patches, including data sources and processing.

Variable type	Variable class	Original variables	Data type	Map original resolution (m)	Analyses	Analyses 2	Derived variable	Source original data
Abiotic	Topographic	Elevation (DEM)	Raster	90	Derivation of two topographic variables using ArcGIS 10.1	-	*Aspect*	(1)
							*Slope*	
	Climatic	Monthly maximum temperature (°C)	Raster	230	Derivation of 19 bioclimatic using dismo package in R version 3.1.2 (6)	Principal Component Analyses (PCA)	*Climate 1 (Axis 1)*, *Climate 2 (Axis 2)*	(2)
		Monthly minimum temperature (°C)						
		Total monthly precipitation (mm)						
	Edaphic	Kw (erodability factor)	Vector		-	Principal Component Analyses (PCA)	*Soil1 (Axis 1)*, *Soil2 (Axis 2)*	(3)
		AWC (available water content)						
		Bulk density						
		Clay content						
		Cation exchange capacity (CEC)						
		pH						
		Percent organic carbon						
		Percent inorganic carbon						
Biotic	Land cover	Land cover (combination land use, climate, and geology)	Raster	30	Reclassification of land cover classes [High urban density, Low urban density, Pasture/Agriculture, Forest age 1 (14–23 yr), Forest age 2 (24–36 yr), Forest age 3 (37–53 yr), Forest age 4 (64–77 yr)]	Assignment of new classes to each of 7 levels of disturbance from 1 (high urban density) to 7 (forest age 4)	*Majority* (M)	(4)
							*Variety* (V)	
							*Range* (R)	

Reference: (1) USGS (www.seamless.usgs.gov); (2) Daly et al. 2003; (3) National Cooperative Soil Survey—USDA Natural Resources Conservation Service (http://ncsslabdatamart.sc.egov.usda.gov and http://websoilsurvey.sc.egov.usda.gov); (4) Helmer et al. 2008.

We used neighborhood functions on the derived variables to represent our three scales of analyses. In doing so, we chose overlapping windows of three sizes [2x2-180 m^2^; 3x3-270 m^2^, and 4x4-360 m^2^) and applied the *Mean* function to the climate (*Climate1* and *Climate2*) and soil (*Soil1* and *Soil2*) maps, and the *Majority*, *Variety*, and *Range* functions to the land use map. The three new land-use variables provide information about the dominant land-use type (*Majority*), the variability of land-use types (*Variety*) and degree of disturbance (*Range*) within a given neighborhood. *Range* can take a value between 0–6, where a value of 0 denotes that all the cells in the neighborhood have the same land cover class, and a value of 6 denotes a neighborhood made up of different land cover classes. Our procedure calculated new values for each pixel based on the values of the surrounding cells without changing the resolution of the maps [51]. We used ArcGIS 10.1 and the *raster* package in R version 3.1.2 to run the neighborhood analysis and extract the biophysical variables for each vine patch.

### Data analysis

For each patch we calculated species richness, diversity (Shannon -Weaver index, *H*) and evenness (Pielou’s *J*) [52] using both vine abundance metrics (*A*_*s*_ and *D*_*s*_). We visually inspected relationships among the five dependent (i.e., Richness, Diversity_As_, Diversity_Ds_, Evenness_As_ and Evenness_Ds_) and the eight independent (e.i., *Aspect*, *Slope*, *Climate1*, *Climate2*, *Soil 1*, *Soil 2*, *Variety and Range*) variables to determine the need to introduce polynomial terms to account for non-linear relationships (Table 1). First, we ran stepwise multiple regressions models to explore the relationships between vine richness, diversity, and evenness with the biotic and abiotic variables representing each of the three scales of analyses. Second, the insights gained from these models led us to build full global models that included interactions and quadratic terms that were used as input in *MuMIn*’s dredge function in R 3.1.2. The dredge function generates a set of models with all the possible combinations of the variables present in the global model and compares them based on their Akaike information criterion (AIC). Model selection was based on AIC values, but also took into consideration model complexity and R^2^ values of the resulting models when the difference in AIC values between models was small (Table B in S1 File) [53]. The selected set of models included significant interactions and polynomial terms, thus following suggestions by Schielzeth [54] we centered and standardized the input variables.

To examine the variation in vine species composition among patches across our study region we combined three approaches. First, we ran a hierarchical cluster analysis on a *site x species* matrix that used the Bray-Curtis dissimilarity index and Ward clustering method [55]. The cluster analysis was followed by an indicator value analysis that identified indicator species for each cluster. Second, we ran non-metric multidimensional scaling ordinations (NMDS) on the *site x species* and the *site x functional group* matrices using both vine abundance metrics (*A*_*s*_ and *D*_*s*_). Also, we used the *site x biophysical variables* (using the derived variables in Table 1.) matrices to examine the correlation between these variables with the ordination axes. We used the Bray-Curtis dissimilarity index in all NMDS ordinations [55]. Finally, we used Veech’s [56] approach to examine species co-occurrences. The *vegan* and *standard* R packages were used to estimate the diversity metrics, conduct the hierarchical cluster analysis, and the NMDS ordination, whereas the *indicspecies* and *cooccur* R packages to run the indicator value and species co-occurrence analyses, respectively.

## Results

A total of 49 vine species were recorded in the 51 patches sampled. Seventeen (35%) species belong to the Fabaceae, eight (16%) to the Convolvulaceae, four (8%) to the Cucurbitaceae, and the remaining 20 species belong to 13 other families (Table C in S1 File). A classification of vine species by climbing mechanism revealed that 35 species (71%) were twiners, nine (18%) used tendrils, and the remaining five (11%) used aerial roots, a scandent or sarmentous mode of climbing. Similarly, a classification by geographic origin showed that 29 species (59%) were native and 20 (41%) were alien.

**Fig 2:**
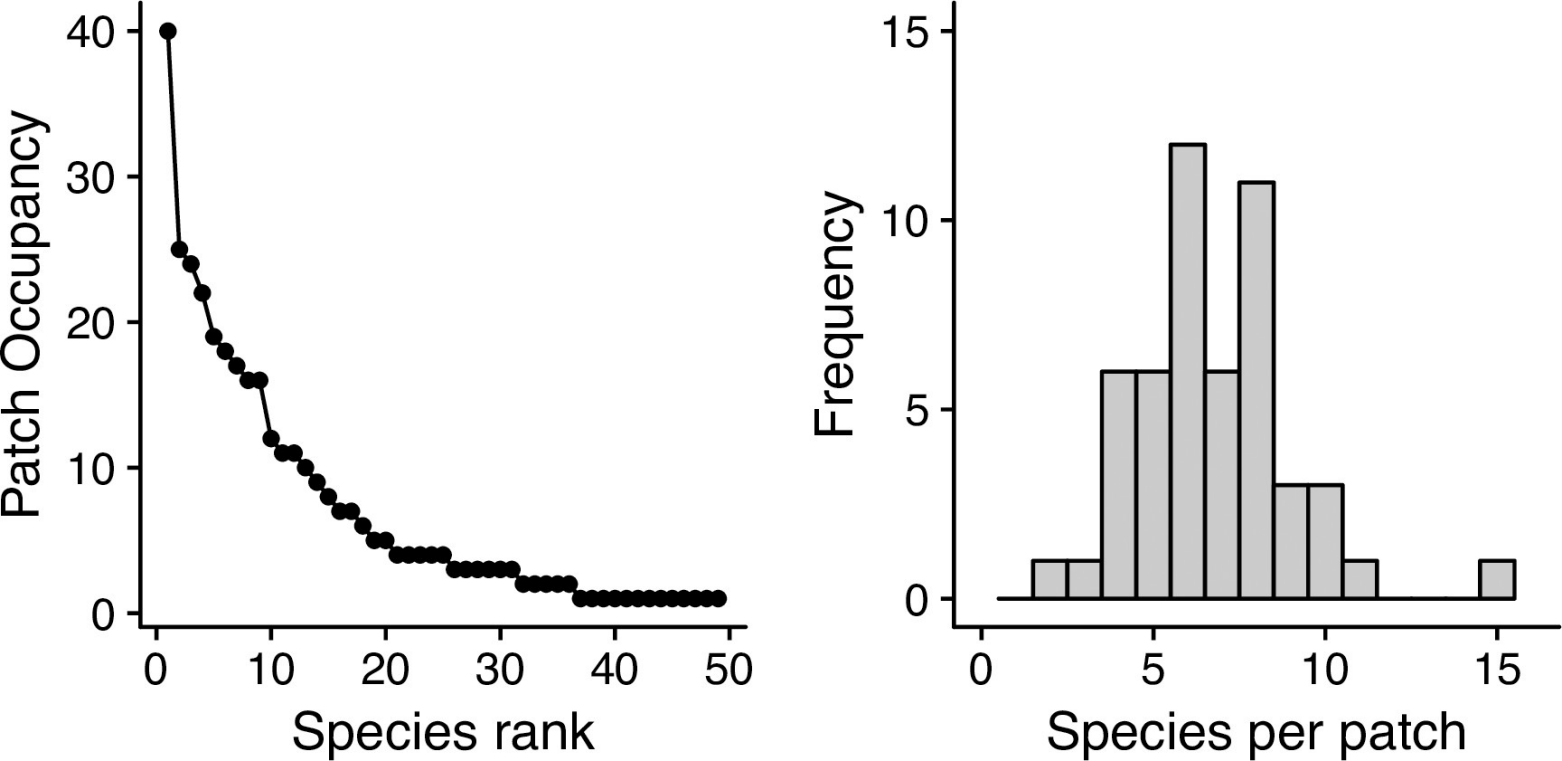
Vine patch diversity. Vine species a) rank-patch occupancy and b) patch richness plots.

A rank-patch occupancy analysis showed that four (8%) species were very common (present in ≥ 20 vine patches), 14 species (28%) were common (present in 6–19 vine patches), and 31 species (63%) were rare (present in 1–5 vine patches). Among the former, *Mikania micrantha* was present in 40 out of the 51 patches, whereas *Ipomoea alba*, *Pueraria phaseoloides*, and *Cissus verticillata*, in 25, 24, and 22 patches, respectively (Fig 2A; Table C in S1 File). All but one of these species (*C*. *verticillata*) were twiners, and all but one species (*P*. *phaseoloides*) were native. In contrast, among rare species roughly half of the species were twiners and the remaining half used tendrils.

### Multi-driver and multi-scale assessment of vine alpha diversity

Mean species richness, diversity, and evenness of vine patches was 6.7±2.3, 1.46±0.41, and 0.78±0.13., respectively (Fig 2B). All models predicting vine patch richness, diversity, and evenness were significant; all but one of the models using vine relative abundance (*A*_*s*_) had slightly greater explanatory power than those using vine relative density (*D*_*s*_). The explanatory power of the models differed with scale and the models varied in terms of the abiotic and biotic variables that were retained (Table 2). The multiple regression models predicting species richness (*R*^*2*^ = 0.12–0.30) had lower explanatory power than those predicting species diversity (*R*^*2*^ = 0.27–0.42) and species evenness (*R*^*2*^ = 0.0.26–0.53). Four out of the seven predictor variables, whether alone or in interaction with a second variable, were retained (*P*≤0.1) by at least one of the 15 models (Table 2). *Climate1* was retained by all models, and in all instances, we observed a non-linear or “humped-back” relationship between species richness, diversity, and evenness and this variable. In contrast, landscape *Variety* was retained by the small and medium-scale models, and it correlated negatively with species richness, diversity, and evenness. *Slope* and landscape *Range* were retained by <30% of the models and often were only marginally significant.

**Table 2:** Results of stepwise multiple regressions models predicting species richness, diversity and evenness. Numbers represent coefficients of the abiotic and biotic variables retained by the models.

Variable type	Variable	180 m					270 m					360 m				
		Richness	Diversity_*As*_	Diversity_*Ds*_	Evenness_*As*_	Eveness_*Ds*_	Richness	Diversity_*As*_	Diversity_*Ds*_	Evenness_*As*_	Eveness_*Ds*_	Richness	Diversity_*As*_	Diversit_*Ds*_	Evenness_*As*_	Eveness_*Ds*_
	Intercept	6.75***	1.49***	1.35***	0.78***	0.71***	6.75***	1.46***	1.34***	0.78***	0.71***	7.44***	1.47***	1.34***	0.78***	0.71***
Abiotic	Climate1		0.21***	0.23***	0.07***	0.10***	0.21	0.09†	0.19***	0.08***	0.11***		0.17**	0.20***	0.08***	0.11***
	Climate1^2	-0.68*	-0.17**	-0.18**	-0.03*	-0.04*	-0.7*	-0.18***	-0.19***	-0.004**	-0.06**	-0.38*	-0.19***	-0.19**	-0.004**	-0.06**
	Climate2															
	Soil1															
	Soil2															
	Slope				0.04*	0.03†				0.02					0.02	
Biotic	Variety	-0.73	-0.14†	-0.13*	-0.02†	-0.04*	-0.96*	-0.17**	-0.14**	-0.03*	-0.04*					
	Range	0.56	0.06				0.55	0.04								
	Climate1:Variety		-0.24**	-0.11*												
	Climate1:Range		0.24*				-0.99*	-0.14*								
	Climate1:Slope															
	Climate1^2:Variety						-0.07								
	Climate1^2:Range	-0.71†	-0.13*													
	Climate1^2:Slope				0.04*											
	*P*	0.01	<0.001	<0.001	<0.001	<0.001	0.01	<0.001	<0.001	<0.001	<0.001	0.01	<0.001	<0.001	<0.001	<0.001
	R-squared	0.25	0.46	0.42	0.53	0.50	0.30	0.47	0.39	0.50	0.48	0.12	0.27	0.29	0.46	0.26

Significance level ≤0.001 (***), ≤ 0.01 (**), ≤0.05 (*), ≤0.10 (†).

### Multi-driver and multi-scale assessment of vine beta diversity

**Fig 3:**
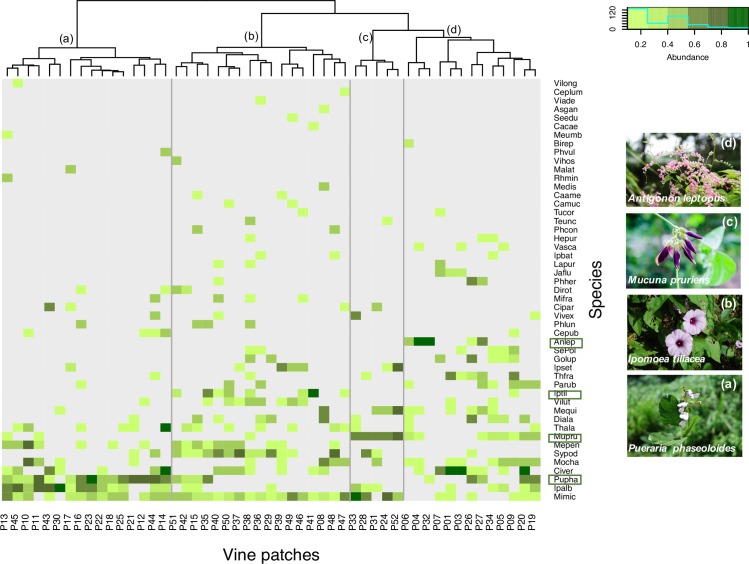
Heat map of vine patches and species abundances. The dendrogram on the top identifies four clusters generated by the hierarchical cluster analysis (a-d; based on Bray-Curtis dissimilarity index and Ward clustering methods). The photos on the right shows the main indicator vine species of the four clusters.

Vine patches were separated into four main clusters (Fig 3; Table D in S1 File). The indicator species analyses identified one indicator species *(P*. *phaseoloides*, *P = 0*.*001)* for Cluster *a* (16 patches), two species (*Ipomoea tiliacea*, *P = 0*.*004* and *Vigna luteola*, *P = 0*.*009*) for Cluster *b* (17 patches), one species (*Mucuna pruriens*, *P = 0*.*001)* for Cluster *c* (5 patches), and lastly four species (*Antigonon leptopus*, *P = 0*.*004*, *Valeriana scandens*, *P =* 0.036, *Jasminum fluminense*, *P =* 0.037, and *Passiflora rubra*, *P = 0*.*036*) for Cluster *d* (17 patches).

**Fig 4:**
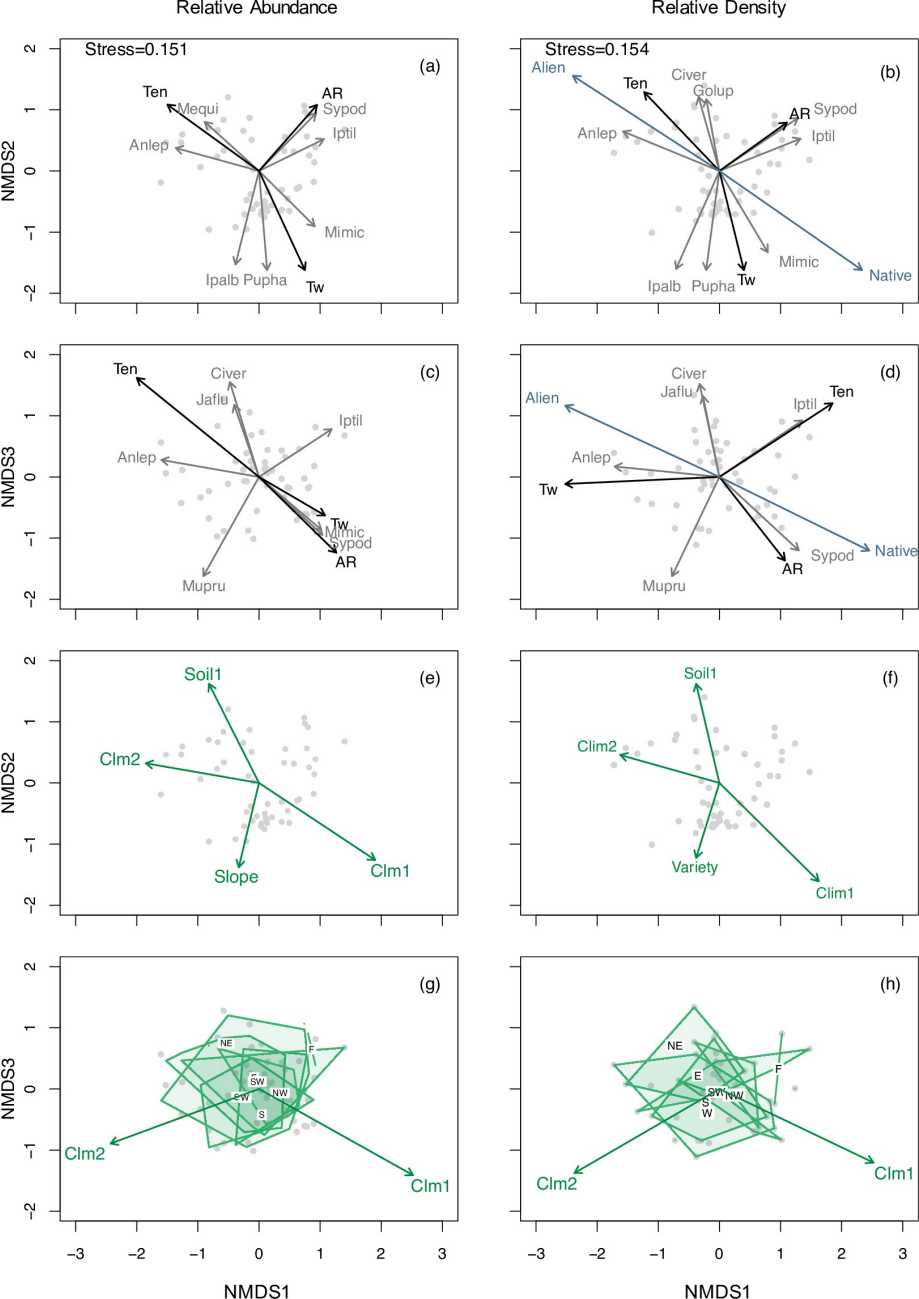
**Non-metric multidimensional scaling-NMDS ordination of vine patches based on species’ relative abundance (*A***_***s***_**; a, c, e, g) and relative density (*D***_***s***_**; b, d, f, h).** A secondary matrix of abiotic and biotic variables is based on medium-scale (270 m^2^) spatial data. In a-d species are represented by grey and functional groups by black vectors; only species and functional groups significantly correlated (α = 0.001 and 0.05, respectively) with the ordination axes are shown. In e-h abiotic variables are represented by green vectors or polygons (categorical; aspect); only the variables that were significantly correlated with the ordination’s axes are shown (α = 0.05). Gray dots are vine patches. The species codes are provided in Table C in S1 File. Functional groups: Te; Tendrils, Tw; Twining, Sc; Scandent/Sarmentous, AR; Aerial roots. Aspect: F; Flat, NE; Northeast, E; East, SE; Southeast, S; South, SW; Southwest, W; West, NW; Northwest.

The NMDS ordinations provided further insights into the relationships among vine patches, as well as the biotic and abiotic factors underlying variation in species composition at the scale of our study. The ordinations yielded slightly different results depending upon the metric used to calculate vine abundance and the scale of the biotic and abiotic variables (Fig 4; Table 3; Figure A in S1 File; Table C in S1 File). The NMDS based on species’ relative abundance (*A*_*s*_) yielded a three-dimensional solution with a stress value of 0.151 (Fig 4A and 4C; Table 3). We identified four groups of vine patches based on the species exhibiting the largest correlation values with the ordination axes. Vine patches were separated along Axis 1 based on the presence and abundance of *M*. *micrantha* (positive correlation; *R*^*2*^ = 0.34), and *Merremia quinquefolia* and *A*. *leptopus* (negative correlation; *R*^*2*^ = 0.29 and 0.41, respectively). Similarly, vine patches were separated along Axis 2 based on the presence and abundance of *I*. *alba and P*. *phaseoloides* (negative correlation; *R*^*2*^ = 0.51 and 0.64, respectively), and *I*. *tiliaceae* and *Syngonium podophyllum* (positive correlation; *R*^*2*^ = 0.304 and 0.36, respectively). Vine patches were separated along Axis 3 of the ordination based on the presence and abundance of *M*. *pruriens* (negative correlation; *R*^*2*^ = 0.52), and *C*. *verticillata* and *J*. *fluminense* (positive correlations; *R*^*2*^ = 0.48 and 0.27, respectively). Climbing mechanism (*A*_*fc*_) provided further insights into the observed differences among vine patches: *tendril climbers* (R^*2*^ = 0.57), correlated negatively, and *aerial roots climbers* positively (R^*2*^ = 0.26), with Axis 1, whereas t*winning* vines correlated negatively with Axis 2 (*R*^*2*^ = 0.53). Similarly, significant correlations between five biophysical variables (medium-scale) and the ordination axes indicate that species vary in their tolerance to biophysical conditions (Fig 4E and 4G; Table 3). *Climate1* was positively (R^*2*^ = 0.29), and *Climate2* (R^*2*^ = 0.20), negatively, correlated, with Axis 1; on the other hand, *Soil1* (R^*2*^ = 0.19), was positively, and *Slope* (R^*2*^ = 0.14), negatively, correlated with Axis 2.

**Table 3:** Results of NMDS ordinations. R-squared values for the correlations between abiotic and biotic variables, including functional traits and geographic origin, with the three first axes of the ordinations using permutations tests (n = 1000).

Abundance Metric	Variable type	Variable	180 m			270 m			360 m		
			NMDS1	NMDS2	NMDS3	NMDS1	NMDS2	NMDS3	NMDS1	NMDS2	NMDS3
Relative species abundance (*A*_*s*_)	Abiotic	Climate 1	0.284**	0.138*		0.286***	0.133*		0.297***	0.125*	
		Climate 2	0.228***		0.015	0.197**		0.183	0.201**		0.022
		Soil 1		0.029	0.019	0.054	0.191**		0.05	0.188**	
		Soil 2	0.045	0.033		0.021		0.002	0.049	0.004	
		Slope		0.139*			0.144*			**0.101†**	0.011
		Aspect	0.220*	0.193	0.148	0.186	**0.212**†	**0.238**†	0.143	**0.210**†	0.203
	Biotic	Majority	0.174	0.146	0.193	0.142	0.118	0.191	0.07	0.073	0.065
		Variety		0.037	0.005		0.083	0.003	0.001	0.026	
		Range		0.003	0.001		0.028	0.002	0.001	0.051	
	Climbing mechanism	Twining	0.132**	0.534***							
		Tendrils	0.571***								
		Aerial Roots	0.263**	0.001***							
		Scandent	0.073								
	Geographic origin	Native	0.019		0.041						
		Alien	0.019		0.041						
Relative Species Density (*D*_*s*_)	Abiotic	Climate 1	0.155*	0.288**		0.302***	0.170*		0.308***	0.171*	
		Climate 2	0.221**		0.043	0.164**		0.049	0.157*		0.046
		Soil 1	0.025		0.019		0.160*			0.157*	0.028
		Soil 2		0.054	0.005	0.022	0.01		0.03	0.047	
		Slope	0.012	**0.103†**		0.009	0.077			0.077	0.012
		Aspect	0.193	0.156	0.138	0.183	**0.220**†	0.240*	0.159	0.159	0.172
	Biotic	Majority	0.19	0.154	0.195	0.242	0.082	0.055	0.072	0.082	0.055
		Variety		0.05	0.005	0.009	**0.095**†			0.025	0.000
		Range		0.041	0.003	0.008	0.028			0.042	0.002
	Climbing mechanism	Twining	0.037	0.519***							
		Tendrils	0.402***	0.590***							
		Aerial Roots	0.368***		0.229**						
		Scandent	0.082	0.023							
	Geographic origin	Native	0.394***	0.155*							
		Alien	0.399***	0.143*							

Significance levels: ≤0.001(***), ≤0.01 (**), ≤0.05 (*), and ≤ 0.10 (†)

The NMDS based on species’ relative densities (*D*_*s*_; stress value of 0.154; Fig 4B and 4D; Table 3; Table C in S1 File) yielded similar results as the one based on species’ relative abundance (*A*_*s*_) with three exceptions. First, we observed slight changes in species’ correlation values (Table C in S1 File). Second, vine patches were separated along Axis 2 based on the presence and abundance of *M*. *micrantha* (negative correlation; *R*^*2*^ = 0.34), and *Gouania lupuloide* and *C*. *verticillata* (positive correlation; *R*^*2*^ = 0.21 and 0.48, respectively). Third, geographic origin (*D*_*fg*_) explained part of the observed variation among patches: native species exhibited a positive correlation (*R*^*2*^ = 0.39) and alien a negative correlation (*R*^*2*^ = 0.40) with Axis 1 (Fig 4B and 4D; Table 3). One additional biophysical variable, namely landscape *Variety*, correlated negatively with Axis 2 (Fig 4F; Table 3).

**Fig 5:**
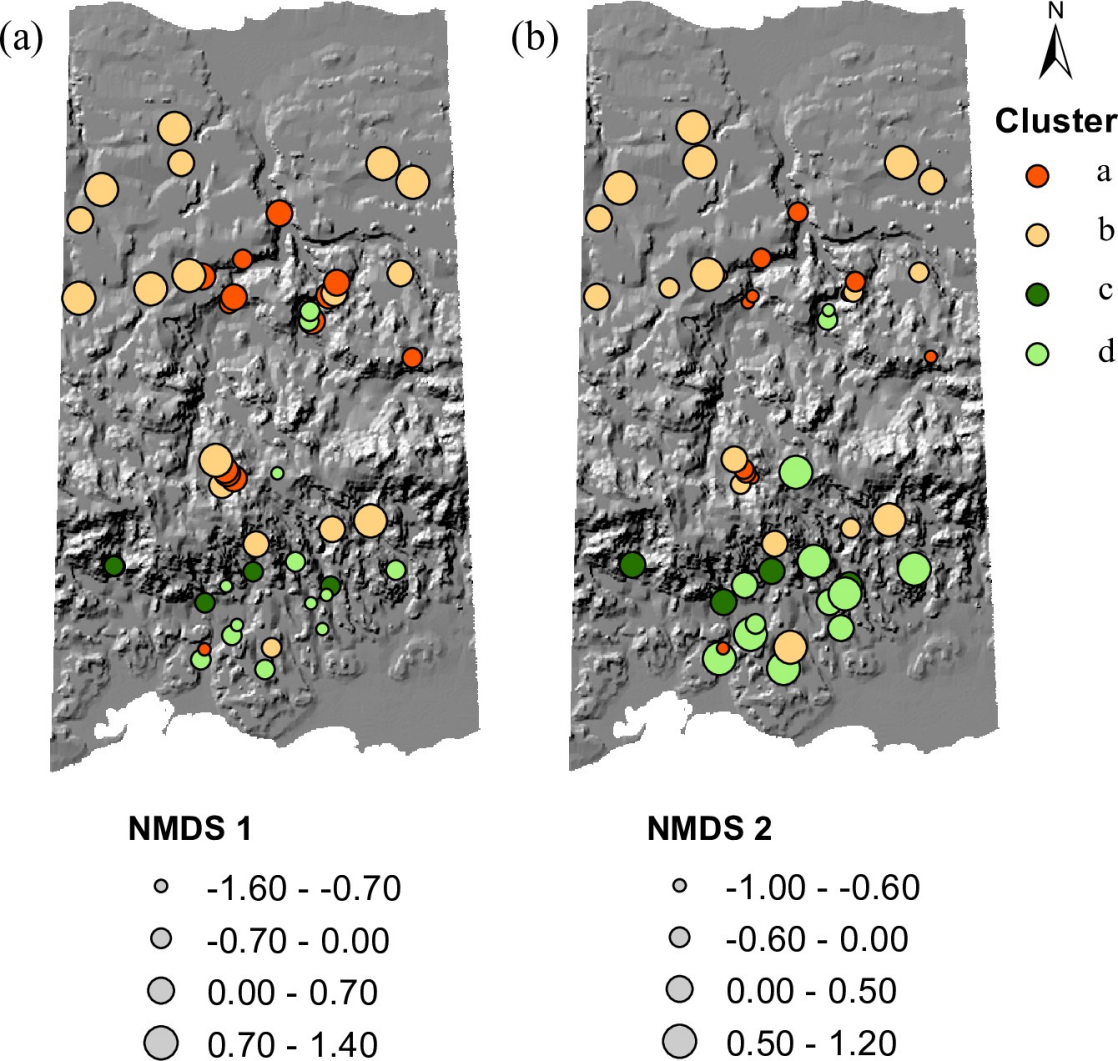
**Spatial distribution of NMDS scores for the (a) first and (b) second NMDS ordination axes.** The colors show each vine patch grouped by cluster according to the hierarchical cluster analysis.

Mapping the ordination scores and cluster affiliations in geographic space showed that patches with positive values along Axis 1 were found mostly in the north and the central mountainous region of our study area, whereas vine patches with negative values were mostly restricted to the southern side of the central mountains (Fig 5). On the other hand, patches with negative scores along Axis 2 were mainly concentrated in the central mountainous region (Fig 5). Species falling within Cluster *a* were concentrated in the central mountainous region of our study area, while species in Cluster *c* and *d* were almost exclusively found in the southern part. Finally, species in Cluster *b* were widely distributed in our study area.

The cluster analyses as well as the ordinations suggested associations among species that were further evaluated. Of a total of 1,176 possible species’ co-occurrences, 30 (2.5%) were significantly non-random (Figure B in S1 File). Eleven of these non-random co-occurrences were classified as positive, whereas 17 as negative. If we focus on the species, five of these always showed negative co-occurrences with other vine species, whereas six species showed only positive co-occurrences; the remaining 10 species exhibited both positive and negative co-occurrences. Species found to always form negative co-occurrence pairs included *C*. *verticillata*, *I*. *tiliaceae*, *M*. *micrantha*, *Thunbergia fragans*, *T*. *alata*, whereas species found always in positive co-occurrence pairs included *Dioscorea alata*, *D*. *rotundata*, *I*. *setifera*, *Melothria pendula*, *P*. *rubra*, *and S*. *podophyllum*.

## Discussion

Focusing on vine communities, we asked three questions aimed at understanding patterns of alpha and beta diversity as a function of abiotic and biotic variables and scale, as well as patterns of vine co-occurrences, in a region underlain by complex environmental gradients. One abiotic (*Climate1-summarizes the* largest variability among temperature and precipitation variables) and one biotic (landscape *Variety -*. expresses landscape heterogeneity within a neighborhood) variable were consistently important in explaining variation in species richness and diversity at small and medium scales, and species composition at medium scales. In contrast, only abiotic variables were important in explaining variation in species richness, diversity, and composition at large scales. Significant patterns of co-occurrences, some positive and some negative, suggest that interactions among parasites contribute to the organization of vine communities, yet the scales at which they occur are not readily identifiable by this study. Altogether, our results indicate that abiotic factors in tandem with *host-parasite* and *parasite-parasite* interactions are important in the assembly of vine communities but depending upon scale.

### Multi-driver and multi-scale assessment of alpha diversity

One abiotic variable (*Climate1*) was an important predictor of vine richness, diversity, and evenness at all scales, whereas one biotic variable (landscape *Variety*) was an important predictor of all three metrics at small and medium scales. In all instances, these three variables exhibited a “humped-back” relationship with *Climate1* (Table A in S2 File). Thus, in our study area, richness, diversity, and evenness were highest in areas with intermediate temperatures and precipitations, and lowest in hot/dry and cool/wet areas. These results contrast with works on lianas [33, 34, 57] and other parasites [58] showing that parasite richness varies linearly and positively with climatic variables (but see [59]). Work with other taxa or functional groups has shown both patterns, i.e., species richness may vary linearly [60] or non-linearly [61] with temperature, precipitation, and evapotranspiration. At least two non-mutually exclusive hypotheses may explain the observed “humped-back” relationship. First, vines are directly influenced by *Climate1*, and only a small subset of species thrive at the extremes of *Climate1* (see below). Second, vine hosts are directly influenced by *Climate1* and vines indirectly through host-parasite interactions.

In contrast to *Climate1*, landscape *Variety*—the number of land use types within our windows of analyses—was linearly and negatively related to vine patch richness, diversity, and evenness. These results also differ from those reported for lianas [37, 38] and other parasites [9, 62, 63] describing a positive relationship between parasite diversity and host or habitat diversity. Three non-mutually exclusive hypotheses may help interpret these results while offering general insights into *vine-vine* and *host-parasite* interactions at regional scales. A first hypothesis states that land use legacy or landscape memory arrests vine succession [64]. Puerto Rico is among several regions worldwide where the large-scale abandonment of agriculture [46, 65] resulted in a mosaic of old-growth fragments, second-growth forests of various ages, areas devoted to small-scale agriculture, cattle ranching, and suburbs. In these post-agricultural landscapes native and non-native vines blanket crops, second-growth vegetation, and forest edges ([42, 44], e.g., [66]). The intentional introduction of non-native vines, repeated sowing of vine seeds, and soil disturbance [47] could have favored the spread of a small group of vines in areas that were formerly devoted to agriculture. Thus *vector-vine* interactions may explain discrepancies between our observations and those made by others.

The other two hypotheses involve the data and land-use metrics that we used. Specifically, fine-scale host heterogeneity is not captured by our landscape metrics. This fine-scale heterogeneity may be represented by two types of hosts, namely other vines (*parasite-parasite* interactions) and other plants taxonomically and/or functionally diverse. In fact, vines and lianas can serve as hosts to species with similar climbing habits and even contribute to their spread [41, 42]. Our last hypothesis poses that heterogeneous landscapes may limit the dispersal and establishment of vine species, especially in areas where multiple land uses occur together in relatively close proximity, effectively limiting the total area covered by each land use class. In this case, *host-vine* interactions may result in a decrease in species richness and diversity.

The recent passage of Hurricane Maria through Puerto Rico is an important reminder of the likely role that stochastic factors, and hurricanes, in particular, may have in the assembly of vine communities [42, 67]. Informal aerial and road surveys through our study area in the aftermath of Maria showed that most of the observed greenery amidst widely defoliated areas corresponded to patches of vines and to a lesser extent grass and that the area covered by vines was likely to be larger than that estimated by our previous work [44]. Prior to Hurricane Maria, people in the countryside commonly mentioned an increase in vine cover associated with previous hurricane activity. Thus, in these landscapes, a fraction of the unexplained variation in our data could result from the legacy of hurricane activity.

### Multi-driver and multi-scale assessment of beta diversity

The clustering and ordination analyses yielded complementary results, yet the latter provided greater insights into the processes underlying patterns of vine beta diversity, as well as the mechanisms underlying vine proliferation. First, a small number of vine species with high abundances contributed to the separation of patches in ordination space. Second, in contrast to our analyses on alpha diversity, a larger number of abiotic variables (*Climate1*, *Climate2*, *Soil1*, and *Slope*) were important to explain variation in species composition; only one biotic variable (*Variety*) was significantly correlated with the ordination axes (medium-scale, *D*_*s*_).

Seven species with the highest correlation values separated vine patches into four groups, all of them including at least one twining species. One group was dominated by one native (*M*. *micrantha*), and the other three by three pairs of native-alien species (*M*. *quinquefolia-A*. *leptopus*, *I*. *alba-P*. *phaseoloides*, and *I*. *tiliaceae-S*. *podophyllum*). Interestingly, these three natives were in the Convolvulaceae family. The dominance of these particular species, as well as the biophysical conditions correlating with vine patches along the first two axes of the ordination, raises questions about the role of *vine-vine* interactions in the assembly of these communities and the traits that may favor them under contrasting biophysical conditions. All these vine species reproduce vegetatively and exhibit medium to very high germination rates. All but one species (*S*. *podophyllum*) show high growth rates, at least three of these species (*I*. *tiliacea*, *A*. *leptopus*, and *P*. *phaseoloides*) have storage roots, and at least two species (*P*. *phaseoloides* and *M*. *micrantha*) are known to alter soil nutrient conditions (Table E in S1 File). These suites of traits suggest a strategy whereby the long-term persistence of these vines may translate into a great potential for blanketing their hosts (i.e., virulence) as shown in other host-parasite systems [68]. Most vine species, however, were found growing in a mixture with other vine species and we predict that these species may have a different combination of traits. Moreover, we predict that positive *vine-vine* interactions may drive the persistence of these vine patches. Unfortunately, there is little information for most of the vine species recorded in our study region.

### Vine communities and global change

In general, the postulated responses of parasites to global changes include range shifts, the decline or extinction of a significant fraction of species and the proliferation of a small fraction of these species [58, 69]. The mechanisms underlying these responses are diverse and complex, operating both at host and parasite levels. Host traits predicting parasite susceptibility to global change include host functional group and host size, whereas parasite traits include degree of parasite specialization, capacity for switching hosts, parasite mode of transmission, persistence, and virulence [58].

Vines, a special group of parasites, are likely to respond in similar ways to global changes. Yet when we consider compact vine communities, like ours, that tend to persist in time and space, we can expect changes in species and functional composition, and/or the expansion of vine patches. This has important implications for the management and conservation of post-agricultural landscapes. We showed that a subset of land use and climatic variables explained a number of community attributes along a complex environmental gradient. Thus, not only climatic changes but also social and economic shifts that influence landscape management may impact vine communities. Climate change can directly influence the growth and distribution of vine species and indirectly may influence landscape management decisions. In the context of this work, this maps directly onto host attributes. Social and economic shifts can influence vine communities independently of climate by facilitating propagule movement, augmenting propagule pressure, and facilitating the establishment of vine communities in certain areas. Our work also showed the presence of multiple groups of co-occurring vine species that differed in terms of their distribution along our environmental gradient. The existence of these groups may ensure the persistence of vine patches under changing environmental conditions. Vine community studies like ours are rare but can be critical in the development of management plans to deal with the spread and expansion of these vine communities. Furthermore, one important contribution of our work is the recommendation to change the scale of the management approach. Instead of a focus on individual species at specific sites, vine management needs to take into consideration both the interactions among multiple vine species within the communities and the interactions among communities in the landscape.

## Conclusion

Both abiotic and biotic variables were important to explain vine species richness, diversity, and composition. However, the identity and importance of these variables varied with scale. Significant patterns of species co-occurrences suggest that parasite-parasites interactions contribute to the organization of vine communities, yet the scales at which they occurred cannot be identified in this study. Altogether, our results indicate that the combined effect of abiotic and biotic factors are important in the assembly of vine communities but that they are scale-dependent. The increasing vine cover in different regions around the world and their reported negative impacts on hosts has made vines a group of conservation concern. Our results may be critical to understand vine proliferation and help design management strategies for their control.

## Supporting information

S1 File Non-metric multidimensional scaling-NMDS ordination of vine patches based on species’ relative abundance and species relative density at small and large scales (Figure A). Results from co-occurrence analysis (Figure B). Bioclimatic and edaphic variables characterizing the study area (Table A). The top three multiple regressions models, based on AIC values, predicting species richness, diversity and evenness based on biotic and abiotic variables for each scale of analysis (Table B). Vine species recorded in vine patches (Table C). Characteristics of vine patch clusters (Table D). Trait The top three multiple regressions models, based on AIC values, predicting species richness, diversity and evenness based on biotic and abiotic variables for each scale of analysis. The models were produced by the dredge function of the MuMIn package in R 3.1.2. In bold are the models discussed in our study. s of vine species with high correlations along Axes 1 and 2 of NMS ordination (Table E).
(DOCX)

S2 File Principal Component Analysis of bioclimatic variables including loadings for each variable at the three scales of analysis (Table A). Principal Component Analysis of edaphic variables including loadings for each variable at the three scales of analysis (Table B).
(DOCX)
