# Supplementary material for: Multi-driver and multi-scale assessment of vine community structure and composition across a complex tropical environmental matrix
Source: PLoS One. 2019 May 10;14(5):e0215274. doi: 10.1371/journal.pone.0215274 (PMC6510454; doi:10.1371/journal.pone.0215274)
Supplement: S1 File — Non-metric multidimensional scaling-NMDS ordination of vine patches based on species’ relative abundance and species relative density at small and large scales (Figure A). Results from co-occurrence analysis (Figure B). Bioclimatic and edaphic variables characterizing the study area (Table A). The top three multiple regressions models, based on AIC values, predicting species richness, diversity and evenness based on biotic and abiotic variables for each scale of analysis (Table B). Vine species recorded in vine patches (Table C). Characteristics of vine patch clusters (Table D). Trait The top three multiple regressions models, based on AIC values, predicting species richness, diversity and evenness based on biotic and abiotic variables for each scale of analysis. The models were produced by the dredge function of the MuMIn package in R 3.1.2. In bold are the models discussed in our study. s of vine species with high correlations along Axes 1 and 2 of NMS ordination (Table E). (DOCX) [file pone.0215274.s001.docx]

Multi-driver and multi-scale assessment of vine community structure and composition across a complex tropical environmental matrix

Diana L. Delgado and Carla Restrepo

**S1**

Relative Abundance

Relative Density

NMDS1

NMDS1

**S1 Fig A.** Non-metric Multidimensional Scaling-NMDS ordination of vine patches based on species’ relative abundance (*A_s_*) at small (a, c) and large (e, g) scales, and species relative density (*D_s_*) at small (b, d) and large (f, h) scales. The abiotic variables are represented by green vectors or polygons (categorical; aspect; only the variables that were significantly correlated with the ordinations axes are shown (α = 0.05). Gray dots are vine patches. Aspect: F; Flat, NE; Northeast, E; East, SE; Southeast, S; South, SW; Southwest, W; West, NW; Northwest.

**S1 Fig B.** Results from co-occurrence analysis. The green color shows pairs of species that co-occur more than expected by chance, while the orange color shows pairs of species that co-occur less than expected by chance. The gray color shows random co-occurrences.

| S1 Table A. Bioclimatic and edaphic variables characterizing the study area. The asterisk (*) denotes variables that were used in the Principal Component Analyses to derive synthetic biophysical variables. | | | |
| --- | --- | --- | --- |
| Variable class | Variable name | Mean (SD) | Variable Id |
| Climatic | Annual mean temperature (°C) | 23.53 (1.39) | Bio 1 |
|  | Mean diurnal range (°C) | 11.46 (0.93) | Bio 2* |
|  | Isothermality | 76.55 (2.18) | Bio 3 |
|  | Temperature seasonality | 126.56 (8.36) | Bio 4 |
|  | Maximum temperature of the warmest month (°C) | 30.65 (1.46) | Bio 5* |
|  | Minimum temperature of the coldest month (°C) | 15.70 (1.38) | Bio 6* |
|  | Temperature annual range (°C) | 14.96 (0.95) | Bio 7 |
|  | Mean temperature of the wettest quarter (°C) | 24.51 (1.19) | Bio 8 |
|  | Mean temperature of the driest quarter (°C) | 21.99 (1.42) | Bio 9 |
|  | Mean temperature of the warmest quarter (°C) | 24.92 (1.35) | Bio 10 |
|  | Mean temperature of the coldest quarter (°C) | 21.94 (1.44) | Bio 11 |
|  | Annual precipitation (mm) | 1773.22 (244.36) | Bio 12 |
|  | Precipitation of the wettest month (mm) | 273.83 (33.72) | Bio 13 |
|  | Precipitation of the driest month (mm) | 57.34 (12.24) | Bio 14 |
|  | Precipitation seasonality | 50.64 (6.59) | Bio 15* |
|  | Precipitation of the wettest quarter (mm) | 710.89 (94.27) | Bio 16 |
|  | Precipitation of the driest quarter (mm) | 196.84 (32.43) | Bio 17 |
|  | Precipitation of the warmest quarter (mm) | 525.46 (139.97) | Bio 18* |
|  | Precipitation of the coldest quarter (mm) | 205.00 (36.44) | Bio 19* |
| Edaphic | Erodibility factor | 0.15 (0.04) | Kw* |
|  | Available water capacity | 0.14 (0.03) | AWC* |
|  | Bulk density | 1.30 (0.11) | Bulk* |
|  | Clay content (%) | 39.27 (9.13) | Clay* |
|  | Cation Exchange Capacity | 29.42 (18.34) | CEC* |
|  | Mean pH | 6.05 (1.15) | pH* |
|  | Organic Carbon (%) | 2.19 (2.27) | OC* |
|  | Inorganic Carbon (%) | 0.92 (1.80) | IC* |

**References**

1. Acevedo-rodríguez P. Vines and Climbing Plants of Puerto Rico and the Virgin Islands. Vol. 51, Smithsonian. 2005. 1-483 p.
2. Croat TB. A Revision of Syngonium (Araceae). Ann Missouri Bot Gard. 1981; 68(4):565–651.
3. Delgado D, Figueroa J, Restrepo C. Using multiple traits to assess the potential of introduced and native vines to proliferate in a tropical region. Ecol Evol. 2016; 6(24):8832–8845.
4. Más EG, Lugo-Torres M de L. Malezas Comunes en Puerto Rico e Islas Virgenes Americanas/Common Weeds in Puerto Rico and the US Virgin Islands. Mayagüez, Puerto Rico: University of Puerto Rico Mayagüez Campus, Natural Resources Conservation Service Caribbean Area; 2013.
5. Space JC, Flynn T, Service USDAF. Report to the Kingdom of Tonga on invasive plant Sspecies of environmental concern. Honolulu, Hawaii USA: Institute of Pacific Islands Forestry; 2001.
6. Wang Y-T, Blessington TM. Growth of four tropical foliage species treated with paclobutrazol or uniconazole. HortScience. 1990; 25(2):202–4.
7. Chen B-M, Peng S-L, Ni G-Y. Effects of the invasive plant *Mikania micrantha* H.B.K. on soil nitrogen availability through allelopathy in South China. Biol Invasions. 2009; 11:1291–9.
8. Department of Agriculture and Fisheries of Queensland. Mikania vine. Department of Agriculture and Fisheries; 2016.

1. Macanawai AR, Day MD, Adkins SW. Seed biology of *Mikania micrantha* in Viti Levu, Fiji. Weed Res. 2018; 58(3):229–38.
2. Shen H, Ye W, Hong L, Cao H, Wang Z. Influence of the obligate parasite *Cuscuta campestris* on growth and biomass allocation of its host *Mikania micrantha*. J Exp Bot. 2005; 56(415):1277–84.
3. Shen S, Xu G, Clements DR, Jin G, Chen A, Zhang F, et al. Suppression of the invasive plant mile-a-minute (*Mikania micrantha*) by local crop sweet potato (*Ipomoea batatas*) by means of higher growth rate and competition for soil nutrients. BMC Ecol. 2015; 15:1–10.
4. Waterhouse DF. Biological control of weeds: Southeast Asian prospects. Vol. 26, Australian Center for International Agricultural Research Monograph. Canberra, Australia: K & B Publications; 1994. 302 p.
5. Willis M, Zerbe S, Kuo Y-L. Distribution and ecological range of the alien plant species *Mikania micrantha* Kunth (Asteraceae) in Taiwan. J Ecol F Biol. 2008; 31(4):277–90.
6. Zhang LY, Ye WH, Cao HL, Feng HL. *Mikania micrantha* H. B. K. in China – an overview. Weed Res. 2004; 44(1):42–9.

1. Atala C, Gianoli E. Induced twining in Convolvulaceae climibng plants in response to leaf damage. Botany. 2008;86:595–602.
2. Austin DF. Moon–Flower (*Ipomoea alba*, Convolvulaceae)—Medicine, Rubber Enabler, and Ornamental: A Review. Econ Bot. 2013; 67(3):244–62.
3. Gunn CR. Seeds of the United States noxious and common weeds in the Convolvulaceae, excluding the genus Cuscuta. Proc Assoc Off Seed Anal. Association of Official Seed Analysts; 1969;59:101–15.
4. McDonald JA. The Systematics and uses of the genus Ipomoea (Convolvulaceae) of Oaxada, Mexico. Vol. M.Sc., Botany. Virignia Polytechnic Institute and State University; 1978.
5. Rambuda TD, Johnson SD. Breeding systems of invasive alien plants in South Africa: does Baker’s rule apply? Divers Distrib. 2004; 10(5‐6):409–16.
6. Torres-Reano G, Alarcon-Bravo L, Frank Austin D, Rojas-Idrogo C, Delgado-Paredes GE. Seed Germination and Seedling Characteristic of ipomoea and Merremia (Convolvulaceae) in Lambayeque (Peru). Pakistan J Biol Sci. 2017; 20(10):507–15.

1. Díaz J, De La Puente F, Austin DF. Enlargement of fibrous roots in Ipomoea section Batatas (Convolvulaceae). Econ Bot. 1992; 46(3):322–9.
2. Martin FW, Jones A. The species of Ipomoea closely related to the sweet potato. Econ Bot. 1972;26(3):201–15.
3. Thaman RR, Fosberg FR, Manner EL, Hassall DC. The flora of Nauru. Washington, DC, USA: National Museum of Natural History Smithsonian Institution; 1994.

1. USDA, National Resources Conservation Service. The PLANTS Database. Team NPD, editor. Greenboro, NC, USA; 2018. Available from: http://plants.usda.gov
2. Bacon PS. The weedy species of Merremia (Convolvulaceae) occurring in the Solomon Islands and a description of a new species. Bot J Linn Soc. 1982;84:257–64.
3. Blanckaert I, Vancraeynest K, Swennen RL, Espinosa-Garcia FJ, Pinero D, Lira-Saade R. Non-crop resources and the role of indigenous knowledge in semi-arid production of Mexico. Agric Ecosyst Environ. Elsevier; 2007; 119(1–2):39–48.
4. Liogier AH. Descriptive flora of Puerto Rico and adjancent Islands. Rio Piedras, P.R.: Editorial de la Universidad de Puerto Rico; 1995.
5. Food and Agriculture Organization of the United Nations. Ecocrop. Vol. 2018. ecocrop.fao.org; 2007. Available from: http://ecocrop.fao.org/ecocrop/srv/en/cropView?id=1824
6. Szott LT, Palm CA, Davey CB. Biomass and litter accumulation under managed and natural tropical fallows. For Ecol Manage. 1994;67:177–90.

1. Telford EA, Office USGP. Tropical Kudzu in Puerto Rico. Circular No. 27. Mayaguez, Puerto Rico: Federal Experiment Station In Puerto Rico; 1947.
2. Burke JM, DiTommaso A. Corallita (*Antigonon leptopus*): Intentional Introduction of a Plant with Documented Invasive Capability. Invasive Plant Sci Manag. 2011; 4(3):265–73.
3. Ernst J, Ketner P, Advies SABC. Study on the ecology and possible control methods of the invasive plant species *Antigonon leptopus* (Corallita or Mexican Creeper). Corallita Pilot Project, St. Eustatius, Netherlands Antilles - Final Report. Aruba; 2007. Available from: http://www.abcadvies.org/rapporten/Coralitta Pilot Project St.Eustatius.pdf
4. Jim CY. Assessing growth performance and deficiency of climber species on tropical greenwalls. Landsc Urban Plan. 2015;137:107–21.
5. Kostikova A, Salamin N, Pearman PB. The role of climatic tolerances and seed traits in reduced extinction rates of temperate Polygonaceae. Evolution (NY). 2014; 68(7):1856–70.
6. Overeem R, Riemens M. *Antigonon Leptopus* (Corallita) on St Eustatius: an Integrated Pest Management approach. Wageningen, The Netherlands: Plant Research International of Wageningen UR; 2018.
