# Supplementary material for: Multi-driver and multi-scale assessment of vine community structure and composition across a complex tropical environmental matrix
Source: PLoS One. 2019 May 10;14(5):e0215274. doi: 10.1371/journal.pone.0215274 (PMC6510454; doi:10.1371/journal.pone.0215274)
Supplement: S2 File — Principal Component Analysis of bioclimatic variables including loadings for each variable at the three scales of analysis (Table A). Principal Component Analysis of edaphic variables including loadings for each variable at the three scales of analysis (Table B). (DOCX) [file pone.0215274.s002.docx]

**Multi-driver and multi-scale assessment of vine community structure and composition across a complex tropical environmental matrix**

Diana L. Delgado and Carla Restrepo

**S2**

Data preparation

*Topographic variables* - We used a DEM to generate two topographic variables, namely *Slope* and *Aspect*; the latter map was reclassified into nine categories (F: flat, N: North, NE: Northeast, E: East, SE: Southeast, S: South, SW: Southwest, W: West, and NW: Northwest). *Slope* is often used as surrogate of soil moisture [1] although it also reflects variation in soil fertility, soil depth, and erosion rates [e.g., 2,3] . *Aspect*, on the other hand, is related to variation in solar radiation and evapotranspiration, even in tropical mountainous terrain [e.g., 4].

*Climatic variables* - Three climatic variables, namely maximum and minimum temperature, and total monthly precipitation [5] were used to generate 19 bioclimatic variables [dismo package in R version 3.1.2; 6; Table 1 of the main text] . Redundant variables were eliminated (Pearson correlations *r* >0.65) and the remaining ones were used in a Principal Component Analysis (PCA; Table A in S2). The first two axes of the PCA explained 80% of the variation in the data and were used as two synthetic climatic variables (*Climate1* and *Climate2*; Table 1 of the main text). Two bioclimatic variables correlated positively [precipitation of the warmest (Bio18) and coldest (Bio 19) quarters] and two negatively [maximum (Bio 5) and minimum (Bio 6) temperatures of the warmest and coldest month, respectively] with Axis 1 (*Climate1*). On the other hand, precipitation seasonality (Bio 15) correlated positively and mean diurnal temperature range (Bio 2), maximum temperature of the warmest month (Bio 5), and precipitation of the coldest quarter (Bio 19) negatively with axis 2 (*Climate2*).

*Edaphic variables* - A total of eight edaphic variables at the taxonomic level of *great group* were extracted from the National Cooperative Soil Survey (Table 1 of the main text) to create raster layers representing each soil variable down to 20 cm depth. Four physical (erodibility factor - Kw, available water content - AWC, bulk density and clay content) and four chemical (cation exchange capacity - CEC, mean pH, percent of organic and inorganic carbon) variables were used to characterize the soils. The eight variables were used in a PCA analysis, and we used the the first two axes (61.9% of the variation in the data; Table B in S2) as two synthetic variables (*Soil1* and *Soil2*; Table 1 of the main text). Variables correlating positively with Axis 1 included Kw, pH, CEC, Organic and Inorganic carbon content (*Soil1*). One variable correlated positively with axis 2 (bulk density), whereas two variables negatively with the same axis (AWC and clay; *Soil2*). All PCAs were preformed using the prcomp function of the *stats* package [7] in R 3.1.2.

*Land use variables* – We used the most recent land use map of the island [8] to generate three variables representing vine’s “host” characteristics. This map depicts urban/built-up lands and forest type, the latter comprising a unique combination of geology, life zone, and age. Given that we had an independent set of quantitative climatic and edaphic variables, we reclassified the land-use map to depict forest types only by age (Table 1 of the main text). The land use map was then reclassified to reflect the degree of disturbance of each land-use class. At one extreme, high urban density received a value of 1, whereas old forest (age 4) a value of 7 (Table 1 of the main text). One important assumption of our work is that forest age represents a good surrogate for host characteristics at the spatial scale of our study.

**References**

1. Dearborn KD, Danby RK, Kikvidze Z. Aspect and slope influence plant community composition more than elevation across forest–tundra ecotones in subarctic Canada. J Veg Sci. 2017; 28(3):595–604.

2. Silver WL, Ostertag R, Lugo AE. The Potential for Carbon Sequestration Through Reforestation of Abandoned Tropical Agricultural and Pasture Lands. Restor Ecol. 2000; 8(4):394–407.

3. Novara A, Pisciotta A, Minacapilli M, Maltese A, Capodici F, Cerdà A, et al. The impact of soil erosion on soil fertility and vine vigor. A multidisciplinary approach based on field, laboratory and remote sensing approaches. Sci Total Environ. 2018; 622–623:474–80.

4. Méndez-Toribio M, Ibarra-Manríquez G, Navarrete-Segueda A, Paz H. Topographic position, but not slope aspect, drives the dominance of functional strategies of tropical dry forest trees. Environ Res Lett. 2017; 12(8):85002.

5. Daly C, Helmer E, Quiñones M. Mapping the climate of Puerto Rico, Vieques and Culebra. Int J Climatol. 2003; 23:1359–81.

6. Hijmans RJ, Phillips S, Leathwick J, Elith J. dismo: Species distribution modeling [Internet]. 2016. p. R package version 1. 0-15. Available from: https://cran.r-project.org/package=dismo

7. R Core Team. R: A language and environment for statistical computing [Internet]. Vienna, Austria: R Foundation for Statistical Computing; 2015. Available from: R Foundation for Statistical Computing, Vienna, Austria

8. Helmer EH, Brandeis TJ, Lugo AE, Kennaway T. Factors influencing spatial pattern in tropical forest clearance and stand age: Implications for carbon storage and species diversity. J Geophys Res. 2008; 113:1–14.
